# 2015-another step along the road in a 40-year journey...

**DOI:** 10.1590/S1806-37132015000100001

**Published:** 2015

**Authors:** Rogério Souza

**Affiliations:** Brazilian Journal of Pulmonology, Editor-in-Chief of the Brazilian Journal of Pulmonology

This is a special year for the Brazilian Journal of Pulmonology (BJP); in October, the BJP will turn 40.^(^ 1 ^)^ Dates such as this are particularly significant, given that they lead us to reflect on many of the aspects related to the existence of the BJP: its history; its current relevance; and its prospects.

The BJP was founded in 1975, and the fact that it continues its journey today is undoubtedly due to the continuous efforts of numerous colleagues who, directly or indirectly, have sought to make the Journal function as a great common denominator linking all members of the respiratory medicine community. This process has been spearheaded by a group of brave editors whose efforts have led the BJP to great achievements: Manoel Lopes dos Santos (1975-6); Bruno Carlos Palombini (1976-8); Carlos Frazzatto Junior (1978-82); José Roberto de Brito Jardim (1982-6); Miguel Bogossian (1986-90); Nelson Morrone (1990-4); Carlos Alberto de Castro Pereira (1994-8); Thais Helena Abrahão Tomaz Queluz (1998-2002); Geraldo Lorenzi Filho (2002-4); José Antônio Baddini Martinez (2004-10); and, most recently, Carlos Roberto Ribeiro Carvalho (2010-4), all of whom have contributed to making the BJP the most important scientific journal in the respiratory field in Latin America and one of the most important scientific journals in Brazil. Such a feat gives us a measure of the responsibility we all have to our Journal henceforward.

In recent decades, the BJP was indexed for SciELO (2002) and, subsequently, for PubMed/MEDLINE (2006), which has greatly increased the visibility of the Journal and therefore the number of articles submitted, particularly after it was indexed for the Thomson Reuters Institute for Scientific Information Web of Science and its first impact factor appeared in Journal Citation Reports.^(^ 2 ^)^ We have recently been penalized by having our impact factor temporarily suspended, a fact that will affect us in the next evaluation.^(^ 3 ^)^ However, we have to bear in mind that this metric associated with scientific publication should be understood within its surrounding context rather than as a single number. In particular, the BJP has two very clear functions: to be a publication of quality, providing a vehicle for scientific production in the field of respiratory medicine, especially in Brazil but also worldwide, and to be the major instrument for the continuing education of pulmonologists in Brazil. For the latter mission, the commitment is to reach into the daily lives of its readers through review articles that reflect the necessities of daily clinical practice in the respiratory field. It is for the dissemination of scientific production that we will use common editorial metrics, such as the impact factor. Such metrics, despite their limitations, allow editorial policy planning, with course changes when necessary, in order to increase the national and international visibility of the Journal, thereby attracting the very best science in the field. To that end, several steps have been taken with a view toward increasing the international exposure of the BJP, among which is the fact that, since 2014, the Journal has been available on PubMed Central, an open archive of the U.S. National Institutes of Health National Library of Medicine, making it possible to access the full-text articles published by the BJP (in English) directly via the PubMed search function.^(^ 4 ^)^

We will now make all articles accepted by the BJP available online ahead of print, in order to maximize the exposure time of our publications. In parallel, all BJP content will be screened for plagiarism with the same tools used by the major scientific journals in the medical field. With this screening, along with the exposure, the trustworthiness of the content of the BJP will also increase significantly.

Although the responsibility of managing the BJP is huge, standing on the shoulders of those who have made the Journal so solid makes the path much smoother. The aforementioned changes are aimed at preparing us for further growth. In addition, another very important change deserves special mention. The board of associate editors of the BJP has grown significantly. In advance, I thank all the editors who have embarked on the project for the next four-year term of editorship of the BJP, which begins now. The participation of such a significant body of researchers in the editorship of the Journal undoubtedly allows a closer relationship between the BJP and major research groups, in Brazil and around the world. It also allows the BJP to play its aforementioned role in the continuing education of respiratory medicine professionals working in Brazil, particularly those who are not affiliated with educational institutions. The selection of the topics of greatest interest to all kinds of readers will come to be shared by a group that is representative of the various fields of study in respiratory medicine, potentially enhancing the scope of the Journal.

The ultimate goal for the next four years is to increase the representativeness of the BJP, not only via its role of disseminating scientific knowledge, which is to be measured by various existing metrics, but also via the role it plays in the education of health professionals in Brazil, which is to be measured by the interest in the different areas of the Journal, on the basis of the number of logins/downloads and requested reprints. Let us hope that the celebrations of and reflections on the last 40 years of BJP history will lead us steadily toward a 2015 full of achievements.
